# Effect of Cyclophilin from *Pyropia Yezoensis* on the Proliferation of Intestinal Epithelial Cells by Epidermal Growth Factor Receptor/Ras Signaling Pathway

**DOI:** 10.3390/md17050297

**Published:** 2019-05-18

**Authors:** Jae-Hun Jung, Jeong-Wook Choi, Min-Kyeong Lee, Youn-Hee Choi, Taek-Jeong Nam

**Affiliations:** 1 Department of Food Science and Nutrition, Pukyong National University, 45, Yongso-ro, Nam-Gu, Busan 48513, Korea; folpll1@naver.com; 2 Institute of Fisheries Sciences, Pukyong National University, Busan 46041, Korea; wook8309@naver.com (J.-W.C.); 3633234@hanmail.net (M.-K.L.); 3 Department of Marine Bio-material & Aquaculture, Pukyong National University, 45, Yongso-ro, Nam-Gu, Busan 48513, Korea

**Keywords:** *Pyropia yezoensis*, cyclohphilin, epidermal growth factor receptor, cell proliferation, recombinant protein

## Abstract

Cyclophilin (Cyp) is peptidyl–prolyl isomerase (PPIase), and it has many biological functions, including immune response regulation, antioxidants, etc. Cyp from red algae is known for its antioxidant and antifungal activity. However, the other biological effects of Cyp from *Pyropia yezoensis* are unclear. In this study, we synthesized Cyp from *P. yezoensis* (pyCyp) and examined its biological activity on IEC-6 cells. First, the MTS assay showed that pyCyp increased cell proliferation in a dose-dependent manner. pyCyp activated the EGFR signaling pathway that regulates cell growth, proliferation, and survival. It induced intracellular signaling pathways, including the Ras signaling pathway. In addition, we observed cell cycle-related proteins. pyCyp increased the expression of cyclin A, cyclin E, and Cdk2, and decreased the expression of p27 and p21 proteins. These results indicate that pyCyp stimulates cell proliferation via the EGFR signaling pathway and promotes cell cycle progression in intestinal epithelial cells. Therefore, we suggest pyCyp as a potential material to promote the proliferation of intestinal epithelial cells.

## 1. Introduction

*Pyropia yezoensis* (*P. yezoensis*) has been cultivated and consumed as food primarily in China, Japan, and Korea [1]. It has very high protein content and contains high-quality proteins among seaweed [2]. Nevertheless, a large amount of *P. yezoensis* is needed for the purification of small amounts of protein. Recently, the development of genetic engineering technology has made it possible to express proteins using genetic recombination technology [3]. Most scientists use *E. coli* for recombinant protein expression because it is fast growing and can be cultured at high density, producing recombinant proteins quickly and at low cost [3]. Cyclophilin (Cyp) is one of the immunophilins. It has peptidyl–prolyl isomerase (PPIase) activity and exists in most organisms and cells, including prokaryotes and eukaryotes [4,5,6]. There are various types of Cyp in human, plants, algae, and so on [4]. It is known that 29 Cyp genes are present in *Arabidopsis thaliana* and 26 Cyp genes are present in the green algae *Chlamydomonas reinhardtii* [7,8]. Although Cyp was initially known to function primarily as an intracellular protein, recent studies revealed that it is secreted by cells in response to inflammatory stimuli [9,10,11]. Extracellular secreted Cyp affects several cells’ biological activity. First, it has antioxidant function for oxidative stresses such as reactive oxygen species (ROS) [12]. Second, it affects immune response and inflammatory stimuli [13]. Third, it is known to promote the growth of some cells, such as vascular smooth muscle cells (VSMC) and endothelial cells [13,14]. In addition, Cyp has several effects such as cell migration, cell cycle regulation, and transcription factor regulation [15,16,17]. Previous studies have confirmed the effect of the peptide from *P. yezoensis* in small intestinal epithelial cell proliferation [18]. Therefore, we used Cyp from *P. yezoensis* (pyCyp) to identify the precise factors involved in small intestinal epithelial cell proliferation. The intestinal epithelium represents a huge surface area that is lined by a monolayer of intestinal epithelial cells (IEC), and it absorbs water and nutrients for maintaining life [19]. When intestinal cells are severely damaged, decreased nutrient, vitamin, and fluid absorption can restrict growth and disrupt hydration and electrolyte balance [20]. Thus, the intestinal epithelial layer displays a strict balance between cell proliferation and cell death in order to maintain the intestinal barrier [19]. Intestinal epithelium is one of the most rapidly proliferating tissues in the body [21]. Its proliferation is influenced by the cell-signaling pathway, in which factors such as epidermal growth factor (EGF) and insulin-like growth factor-I (IGF-I) bind to epidermal growth factor receptor (EGFR) and insulin-like growth factor-I receptor (IGF-IR), respectively [22,23,24]. Receptor tyrosine kinases (RTKs), such as EGFR and IGF-IR, are receptors on the cell surface for signaling many growth factors and hormones [22]. RTK regulates metabolic processes such as normal cell proliferation, differentiation, and growth [23]. RTKs, including EGFR and IGF-IR, are known to induce various forms of cell proliferation when bound to EGF and IGF-I [23,24]. Numerous scientists use various disease models such as the small intestine to study the survival and apoptosis of those cells [25]. In this study, we investigated the effect of the physiological activity of pyCyp in IEC-6 cells, which is a normal small intestinal epithelial cell derived from rat. In addition, we observed their mechanisms of promotional cell proliferation via the activation of EGF and EGF signal pathway proteins when pyCyp was treated in IEC-6 cells. Based on these results, we intend to present the possibility of using the derived protein from *Pyropia yezoensis* as a gastrointestinal protection material.

## 2. Results

### 2.1. Expression and Purification of pyCyp

**Figure 1:**
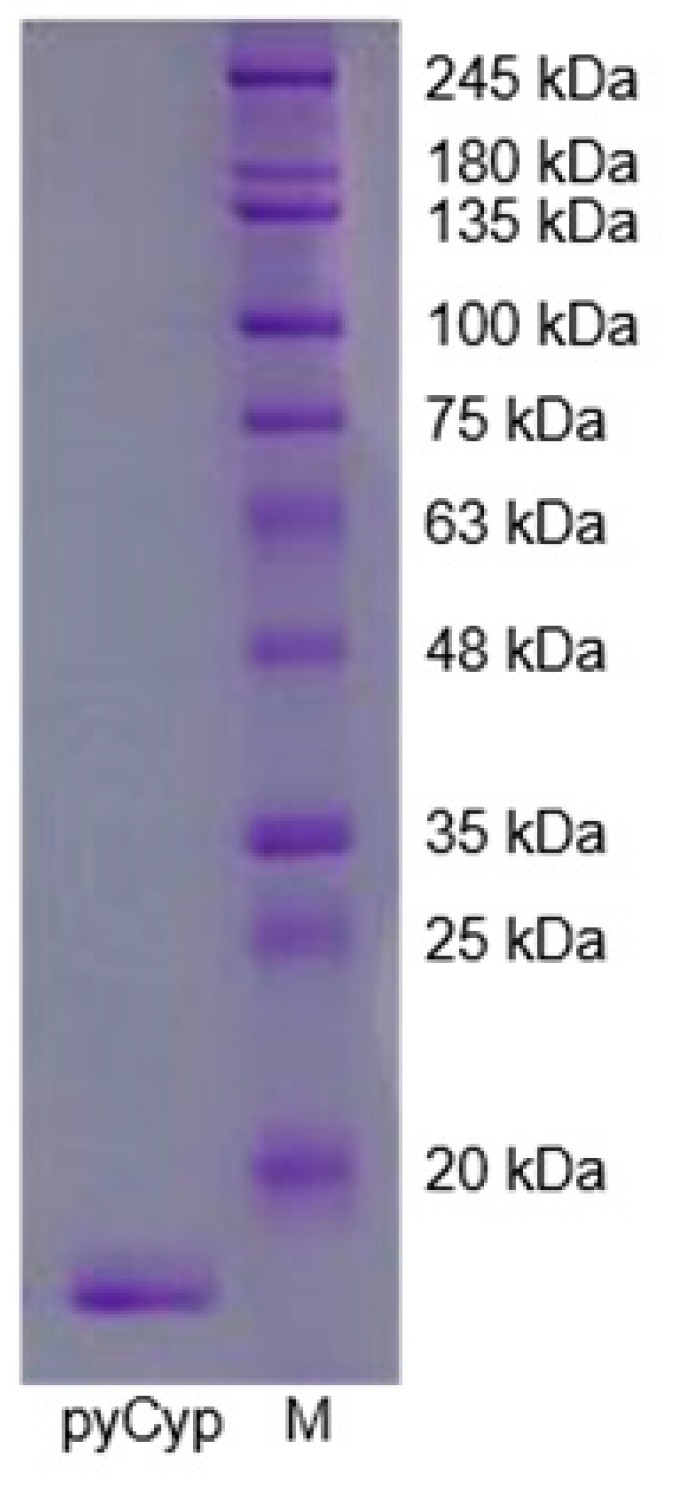
Expression and purification of Cyp from *P. yezoensis* (pyCyp) protein. pyCyp was separated by size exclusion chromatography. One visible band indicates that pyCyp is completely separated.

Sodium dodecyl sulfate polyacrylamide gel electrophoresis (SDS-PAGE) showed a large amount of polypeptide near 20 kDa after the induction of protein expression with isopropyl-β-d-1-thiogalactopyranoside (IPTG). After Ni–nitrilotriacetic acid (NTA) affinity chromatography, the bands at other molecular weights became faint, and only the band of pyCyp near 20 kDa became thick (Figure 1A). It was highly purified with His-tag attached. Next, His-tagged pyCyp was removed with TEV protease and purified once with HiPrep Sephacryl S-200R HR (Amersham Bioscience, Piscataway, NJ, USA). We confirmed one visible band that has a molecular mass of 18 kDa (Figure 1B).

### 2.2. Proliferative Effect of pyCyp in IEC-6 Cells

**Figure 2:**
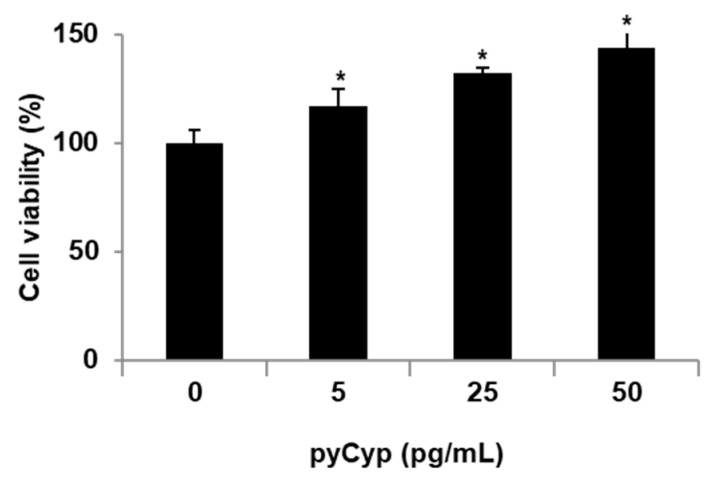
Proliferative effect of pyCyp on intestinal epithelial cell line (IEC-6).

To investigate pyCyp effects in intestinal epithelial cell line (IEC-6), we observed cell viability using MTS assay. After 48 h of pyCyp treatment, cell viability was dose-dependently increased (Figure 2). Especially at 50 pg/mL, viability was increased 40% compared to the control group.

IEC-6 cells were seeded in 48-well plates at 2 × 10⁴ cells/well with medium supplemented with 10% fetal bovine serum (FBS). After incubation for 24 h, cells were serum-starved for 4 h and then treated with pyCyp in the indicated concentrations for 48 h. The results indicate mean ± S.D in triplicate. * *p* < 0.05 versus the corresponding control group and * *p* < 0.05 used to define statistical significance.

### 2.3. Effect of pyCyp Treatment on EGFR Signaling Pathway

**Figure 3:**
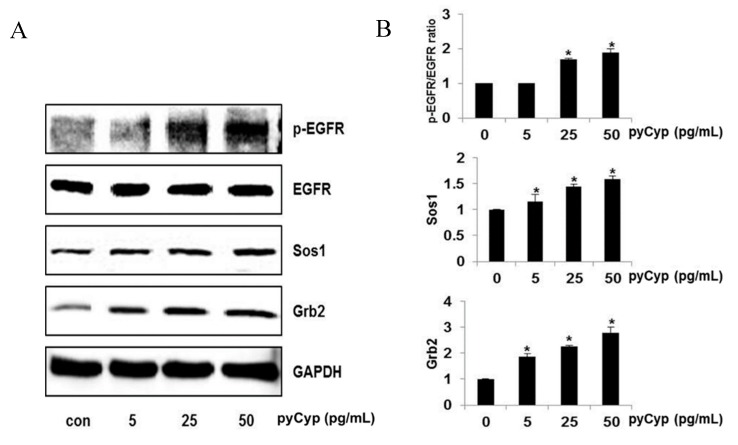
Effect of treatment with pyCyp on epidermal growth factor receptor (EGFR), growth factor receptor-bound protein-2 (Grb2), and son of sevenless (SOS) protein expression levels in IEC-6 cells. (**A**) Proteins were subjected to Western blot analysis. Protein expression levels were increased upon incubation with pyCyp for 48 h. (**B**) Bands were normalized to anti-glyceraldehyde 3-phosphate dehydrogenase (GAPDH) as an internal control. Protein expression or the phosphorylated vs. total protein ratio is graphed. * *p* < 0.05 vs. corresponding control group.

To investigate the mechanism by which pyCyp induces the proliferation of IEC-6 cells, we observed the expression changes of the EGFR-related proteins that are known to be involved in cell proliferation by Western blotting. The targets are phospho-EGFR, Src homology two domain-containingtransforming protein (Shc), growth factor receptor-bound protein-2 (Grb2), and son of sevenless (SOS), which constitute the EGFR in the cell membrane. pyCyp increases the phosphorylation levels of EGFR and the expression of Shc, Grb2, and SOS dose-dependently. These results indicate that pyCyp affects EGFR and induces the expression of related proteins (Figure 3).

### 2.4. Effect of Treatment of pyCyp on the Ras-ERK Signaling Pathway

**Figure 4:**
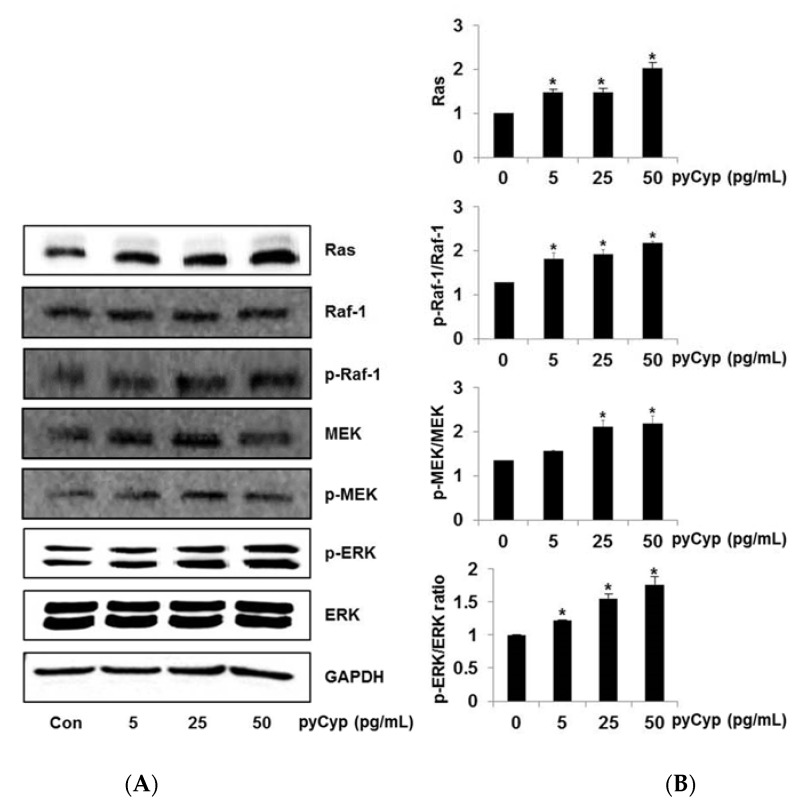
Effects of treatment with pyCyp on Ras, Raf-1, MEK, and ERK protein expression levels in IEC-6 cells. (**A**) Whole-cell extracts were prepared and analyzed by Western blot analysis using anti-Ras, anti-Raf-1, anti-phosphorylated-Raf-1, anti-MEK, anti-phosphorylated-MEK anti-phosphorylated-ERK, anti-ERK, and anti-glyceraldehyde 3-phosphate dehydrogenase (GAPDH) antibodies. (**B**) Bands were normalized to GAPDH as an internal control. Protein expression, or the phosphorylated vs total protein ratio, is graphed. * *p* < 0.05 vs. corresponding control group.

To further investigate the down-signal of EGFR signaling, we examined the Ras–ERK signaling pathway, which is one of the major pathways in EGFR. Its signaling pathway members are renin-angiotensin system (Ras), rapidly accelerated fibrosarcoma-1 (Raf-1), MAPK/ERK kinase (MEK), extracellular signal–regulated kinases (ERK) and so on. The IEC-6 cells were treated with pyCyp for 48 h and analyzed by Western blotting. pyCyp increased the protein expression of Ras, Raf-1, p-Raf-1, MEK, p-MEK, and p-ERK compared with the untreated group. These results mean that pyCyp activates the Ras–ERK signaling pathway following the EGFR signaling pathway (Figure 4).

### 2.5. Effect of pyCyp on Cell Cycle Progression

**Figure 5:**
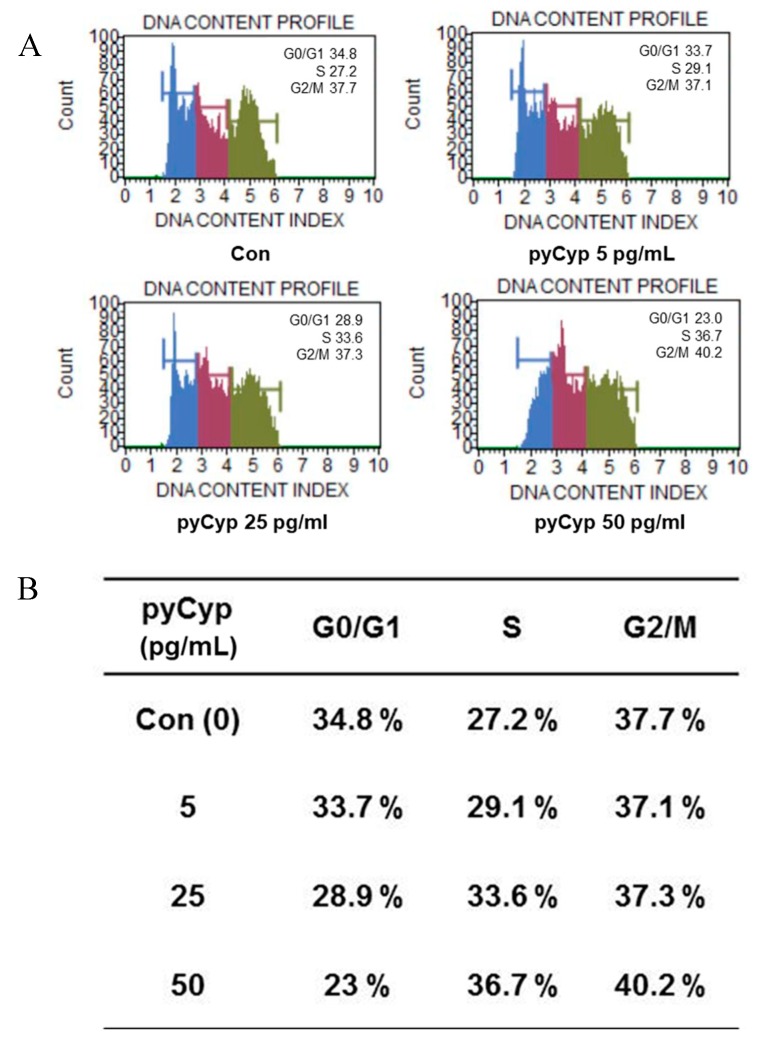
Representative DNA histograms on the cell cycle profile of IEC-6 cells. (**A**) In the dose-dependence experiments, pyCyp was added at each concentration (0 pg/mL, 5 pg/mL, 25 pg/mL, and 50 pg/mL) for 48 h. Cells were harvested and fixed by 70% ethanol for at least more than 3 h. The cell cycle was analyzed by using flow cytometry (Muse Cell Znalyzer). (**B**) Change of cell cycle phase ratio by the pyCyp concentration in IEC-6 cells.

To better understand the phase of cell proliferation, we used flow cytometry to determine the proportion of each phase of the cell cycle. As a result, pyCyp increased the ratio of the S phase in the cell cycle in a concentration-dependent manner. This suggests that pyCyp promotes cell cycle progression by increasing the proportion of the S phase in the cell cycle. This means that pyCyp promotes the proliferation of IEC-6 cells (Figure 5A,B).

### 2.6. Effect of pyCyp Treatment on Cell Cycle-Related Protein

**Figure 6:**
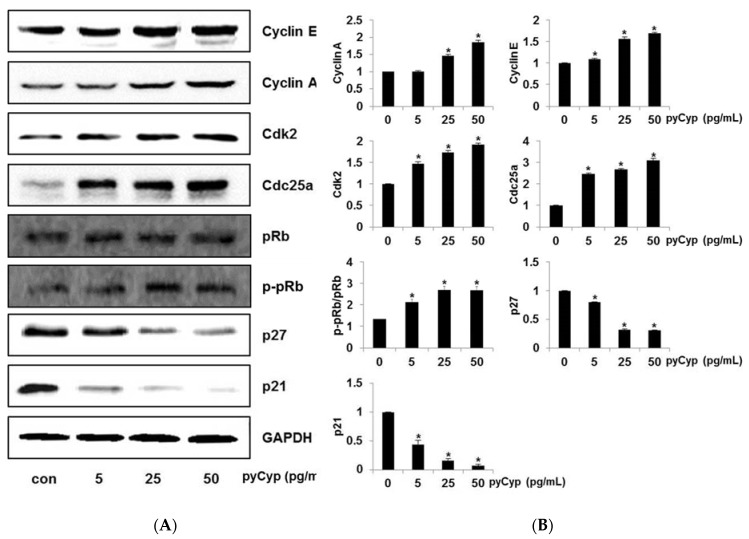
Effect of pyCyp treatment on the levels of cell cycle related proteins in IEC-6 cells. (**A**) Cells were treated with pyCyp after preincubation with SFM for 4 h. Whole cell extracts were prepared and analyzed by Western blotting using anti-Cyclin E, anti-Cyclin A, anti-Cdk2, anti-Cdc25a, anti-pRb, anti-phosphorylated-pRb anti-p27, and anti-p21. (**B**) Bands were normalized to GAPDH as an internal control. Protein expression, or the phosphorylated vs total protein ratio, is graphed. * *p* < 0.05 vs. corresponding control group.

In previous experiments, we confirmed that pyCyp promotes cell proliferation by increasing the proportion of the cell cycle S phase. Thus, Western blotting was performed to observe changes in the expression of factors involved in the cell cycle. The pyCyp was used to investigate the cell proliferation mechanisms that increase cell progression, and we determined the cell cycle-related protein content. In particular, we investigated the factors involved in the S phase of the cell cycle. Expressions of cyclin A, cyclin E, Cyclin dependent kinase 2 (Cdk2), cell division cycle 25a (Cdc25a), retinoblastoma (pRb), and p-pRb, which are involved in the S phase, were increased by pyCyp in a dose-dependent manner. On the other hand, the expression of p27 and p21, which decrease cell cycle progression, decreased in a dose-dependent manner by pyCyp (Figure 6). These results indicate that pyCyp promotes IEC-6 cell proliferation by modulating the cell cycle-related proteins.

## 3. Discussion

Recently, the various physiological activities of algae have been scientifically proven, and they have received much attention in the food, pharmaceutical, and cosmetics industries [2]. Cyp is a ubiquitous protein that is present in all organisms that are involved in a wide range of crucial cellular processes [4,5,6]. It has effects on antioxidants [9], immune response [10], cell proliferation [13], cell migration, cell cycle regulation, etc. [14,15]. Thus, we conducted experiments using the recombinant pyCyp protein to determine the similar biological activity on Cyp from *P. yezoensis*.

In an 3-(4,5-dimethylthiazol-2-yl)-5-(3-caboxymethoxphenyl)-2-(4-sulfophenyl)-2H-tetrazolium (MTS) assay, the viability of IEC-6 cells treated by pyCyp increased in a dose-dependent manner (Figure 2). Then, we examined the mechanism of cell proliferation on IEC-6 cells. Various biological pathways are the major reason for the regulation of cell proliferation. One of the cell proliferation mechanisms, the EGFR signaling pathway, is activated by binding EGF [22,23]. EGFR, one of the RTKs, is known to induce various forms of cell proliferation when bound to EGF. When EGFR is phosphorylated, it binds to proteins such as Sos1 and Grb2 [23]. In this study, pyCyp increases the expression of protein-related EGFR signaling pathways such as phosphorylated EGFR, Grb2, and Sos1 (Figure 3). After this, it activates various signaling pathways including the phosphatidylinositol 3-kinase (PI3K)/protein kinase B (Akt) pathway and Ras–extracellular signal-related kinases (ERK) pathway [23,26]. Sos1 binds to EGFR and acts as a guanine nucleotide exchange factor (GEF), which converts Ras with GDP to Ras with GTP, activating Ras [25]. Activated Ras is involved in a variety of signaling pathways by affecting PI3K, Raf-1, protein kinase C (PKC), etc. [26]. The Ras/ERK biochemical cascade is the important mediator for growth factor-dependent cell survival, proliferation, and differentiation [26,27,28]. Especially, Cyp is known to affect ERK signaling activation [29]. In this study, the expression of the protein-related Ras/ERK signaling pathway increased in a dose-dependent manner (Figure 4). pyCyp promotes cell proliferation by activating the EGFR signaling pathway and its downstream signaling pathway, Ras/ERK.

The cell cycle is the entire cycle from one cell to two daughter cells, including DNA synthesis [29,30,31]. In the G1 phase, the cell increases its supply of proteins for DNA synthesis, increases the number of organelles (such as mitochondria, ribosomes), and grows in size. Next, the cell enters the S phase or stops the cell cycle and enters the G0 phase for differentiation. Sometimes, a cell gets arrested in the G1 phase; hence, it may enter the G0 phase or re-enter the cell cycle [30,31,32]. The cell starts DNA synthesis, and the total amount of DNA in the cell is doubled in the S phase [30,31,32]. When it is completed, all of the chromosomes have been replicated. The G2 phase occurs after DNA synthesis, and is a period of protein synthesis and rapid cell growth to prepare the cell for mitosis [30,32]. In the M phase, the cell separates the chromosomes in its cell nucleus into two identical sets in two nuclei, and finally, the cell is divided into two daughter cells completely [30,32]. The cell cycle is regulated by several molecules such as cyclin and Cdk. They are involved in each phase of the cell cycle, and their functions are different. While cyclin and Cdk promote cell cycle progression, p27 and p21 inhibit cell cycle progression [33]. Cyp also is known to regulate the cell cycle [16]. In this study, we confirmed that the cell cycle was progressed by pyCyp from the G1 phase to the S phase (Figure 5). Cyclin A, cyclin E, Cdk2, and Cdc25a are related in the G1 and the S phases in the cell cycle [32,34]. In addition, pyCyp increases the protein expression of cyclin A, cyclin E, Cdk2, Cdc25a, and pRb, and decreases p21 and p27 (Figure 6). It indicates that pyCyp promotes cell cycle progression from the G1 phase to the S phase.

**Figure 7:**
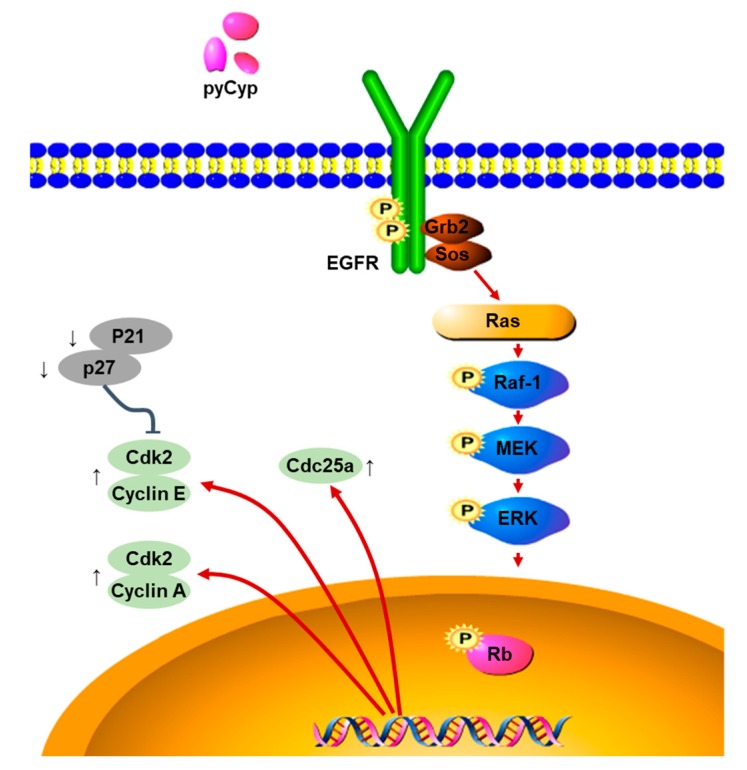
Model of pyCyp protein effects on EGFR signaling in IEC-6 cells.

This result means that pyCyp, which is the recombinant Cyp from *Pyropia yezoensis*, promotes cell proliferation via the EGFR/Ras/ERK signaling pathway. Next, pyCyp promotes cell cycle progression by regulating the G1/S phase and increasing the expression of proteins such as cyclin E, cyclin A, and Cdk2, which are related to the G1/S phases in the cell cycle (Figure 7). This requires more research on the exact mechanism of cell proliferation in the IEC-6 cells, and whether it has an effect on other cell types. However, pyCyp may be a potential candidate for intestinal epithelial cell proliferation, and used to maintain the functioning of the intestinal epithelium.

## 4. Materials and Methods

### 4.1. Recombinant Protein Construction

The open reading frames of the pyCyp gene (residue 1–165; Accession number KJ728870.1) were amplified from the genomic DNA of *P. yezoensis* using the polymerase chain reaction. Forward primer (5′-GGCCATGGCAATGGGGAACCCGCAGGTGTTCTTTGAC-3′) containing the *Nco*I site (underlined) and reverse primer (5′-CACTAACGCCTG ACGCCGCTCGAGATCGAGCTCGGG-3′) containing the *Xho*I site (underlined) were constructed. The DNA fragment was inserted into the *Nco*I and *Xho*I sites of pPROEX-HTA (Invitrogen, Waltham, MA, USA), which is an *E. coli* expression vector. Then, the pPROEX-HTA vector containing the pyCyp gene was transformed into *E. coli* BL21 (DE3).

### 4.2. Recombinant Protein Expression and Cell Lysis

The *E. coli* BL21 (DE3) with the inserted pyCyp gene was cultured in Luria-Bertani (LB) medium containing ampicillin (50 μg/mL) at 37 °C. When the optical density (OD) of the medium reached between 0.6–0.8, 0.5 mM of isopropyl-β-d-1-thiogalactopyranoside (IPTG) was treated at 40 °C to induce protein expression. After 6 hours, the cells were harvested by centrifugation at 8000× *g* and 4 °C for 10 min. The harvested cells were sonicated in a lysis buffer containing 20 mM of Tris (pH 8.0), 150 mM of NaCl, and 2 mM of 2-mercaptoethanol to collect the expressed proteins. The lysates were centrifuged at 25,000× *g* for 30 min, and the supernatant was collected and the next step was carried out.

### 4.3. Recombinant Protein Purification

The supernatant from the previous step was mixed with Ni–NTA affinity resin. The mixture was stirred for 1 h at 4 °C and loaded into the column. An unbound protein was eluted with lysis buffer containing 20 mM of imidazole, and pyCyp was eluted with lysis buffer containing 250 mM of imidazole. To remove the tag of hexa-histidine, the eluate was treated with TEV protease and incubated overnight at room temperature. The pyCyp protein were concentrated using Centriprep (GE Healthcare, Chicago, IL, USA), and purified using HiPrep Sephacryl S-200 HR (GE Healthcare, Chicago, IL, USA) pre-equilibrated with lysis buffer. Purified pyCyp was concentrated to 10 mg/mL in 20 mM of Tris buffer (pH 8.0, 150 mM of NaCl and 2 mM of 2-mercaptoethnol). It was stored frozen at 4 °C until it was used in the experiment, and the concentration was measured by the Bradford assay. The yield was around 10 mg/L of culture medium.

### 4.4. Cell Culture

IEC-6 cells (ATCC CRL-1592) derived from rat were obtained from the American-type culture collection (ATCC; Rockville, MD, USA). Cells were incubated in a humidified 5% CO_2_ incubator at 37 °C in Dulbecco’s modified Eagle’s medium (DMEM) medium supplemented with 10% FBS (HyClone, Inc., South Logan, UT, USA), 100 U/mL of penicillin, and 100 mg/mL of streptomycin. Every 2 days, the medium was replaced.

### 4.5. Cell Viability Assay

Cell viability was determined using a CellTiter 96^®^ aqueous non-radioactive cell proliferation assay (Promega, Madison, WI, USA) according to the manufacturer’s instructions. Cells were seeded in 48-well plates at 2 × 10^4^ cells/well and incubated for 24 h. Attached cells were subsequently treated with pyCyp (0–50 pg/mL) for 48 h. The cells were incubated with 10 μL of MTS solution. After 30 min, the absorbance of each well was measured at 490 nm using a microplate reader (Benchmark microplate reader; Bio-Rad Laboratories, Hercules, CA, USA).

### 4.6. Whole-Cell Protein Lysate Extraction

IEC-6 cells were plated in 100-mm plates and cultured at 37 °C. The cells were subsequently treated with 0 pg/mL, 5 pg/mL, 25 pg/mL, and 50 pg/mL of pyCyp, and incubated for 48 h. Cells were washed with phosphate-buffered saline (PBS) and disrupted on ice in lysis buffer [50 mM of Tris, 5 mM of EDTA, 150 mM of NaCl, and 1% Triton X-100 (pH 7.5)] containing protease inhibitors (1 mg/mL of aprotinin, 1 mg/mL of leupeptin, 1 mg/mL of pepstatin A, 200 mM of Na_3_VO_4_, 500 mM of NaF, and 100 mM of phenyl methylenesulfonyl fluoride; PMSF). The lysates were centrifuged at 12,000× *g*, 4 °C for 20 min, and the supernatant was collected and used for Western blot analysis.

### 4.7. Western Blot Analysis

Protein extracts with consistent concentrations were separated by 6–12.5% SDS-PAGE and transferred to polyvinylidene fluoride (PVDF) membranes (Merck Millipore, Billerica, MA, USA). Membranes were put in 1% bovine serum albumin (BSA) in tris-buffered saline with tween-20 (TBS-T; pH 7.5) containing 0.1% Tween-20, 10 mM of Tris-HCl, and 150 mM of NaCl for 1 h. Next, they were incubated at 4 °C overnight with the indicated primary antibodies [ERK (SC-292838; anti-rabbit), p-ERK (SC-7383; anti-mouse), EGFR (A300-387A; anti-rabbit), p-EGFR (SC-12351, anti-goat), Sos1 (SC-259; anti-rabbit), Grb2 (SC-255; anti-rabbit), Ras (SC-520; anti-rabbit), Raf-1 (SC-227; anti-rabbit), MEK (SC-219; anti-rabbit), cyclin A (SC-271682; anti-mouse), cyclin E (SC-481; anti-rabbit), Cdk2 (SC-163; anti-rabbit), Cdc25a (SC-7389; anti-mouse), p27 (SC-528; anti-rabbit), p21 (SC-397; anti-rabbit), pRb (SC-16670R; anti-rabbit), GAPDH (SC-25778; anti-rabbit)] (Santa Cruz Biotechnology, Inc., Santa Cruz, CA, USA; Bethyl Laboratories, Inc., Montgomery, TX, USA). Secondary antibodies were horseradish peroxidase (HRP)-conjugated goat anti-mouse (SC-2031; Santa Cruz Biotechnology, Inc., Santa Cruz, CA, USA) and anti-rabbit antibody (A90-116P; Bethyl Laboratories, Inc., Montgomery, TX, USA). The signals were detected using SuperSignal West Pico Chemiluminescent Substrate (Thermo Fisher Scientific, Inc., Rockford, IL, USA).

### 4.8. Cell Cycle Analysis

The cells were cultured in 100-mm plates and treated with SFM or each concentration of pyCyp (0 pg/mL, 5 pg/mL, 25 pg/mL, and 50 pg/mL) for 48 h. The cells were harvested by using trypsin and washed with phosphate buffered saline (PBS). Harvested cells were treated with a Muse cell cycle kit (MCH100106; Merck Millipore, Billerica, MA, USA) at 200 μL and stored for 30 min without light. Flow cytometry was conducted using a Muse cell cycle analyzer (Merck Millipore, Billerica, MA, USA) according to the manufacturer’s instructions.

### 4.9. Statistical Analysis

The results were calculated using Microsoft Excel, and are expressed as the mean ± SD. To determine statistical significance, ANOVA was used in SPSS version 18.0 software (SPSS, Inc., Chicago, IL, USA) using one-way analysis of variance followed by Duncan’s multiple range test. A value of *p* < 0.05 was considered to indicate a statistically significant difference.
